# Supplementary material for: Computer-based cognitive interventions in acquired brain injury: A systematic review and meta-analysis of randomized controlled trials
Source: PLoS One. 2020 Jul 9;15(7):e0235510. doi: 10.1371/journal.pone.0235510 (PMC7347178; doi:10.1371/journal.pone.0235510)
Supplement: S2 Table — When more than one test measured the same function, aggregates were produced for the independent meta-analyses. (DOCX) [file pone.0235510.s002.docx]

**S1 Table. Tests used by the included studies to assess the cognitive domains.** **When more than one test measured the same function, aggregates were produced for the independent meta-analyses.**

| **Cognitive domain** | **Study** | **Outcome** |
| --- | --- | --- |
| Visual working memory | Åkerlund et al., 2013 | - Visual Span board forward - Visual Span board backwards |
|  | Cho, Kim & Jung, 2015 | - Visual Span board forward - Visual Span board backwards |
|  | Van de Ven et al. 2017 | - Visual N-back - Corsi blocks task |
|  | Westerberg et al. 2019 | - Visual span board composite |
|  | Yoo et al. 2015 | - Visual span test (The Computerized Neuropsychological Test) |
| Verbal working memory | Åkerlund et al.2013 | - Digit span forward - Digit span backwards |
|  | Cho, Kim & Jung, 2015 | - Digit span forward - Digit span backwards |
|  | Lin et al. 2014 | - Digit span forward and backwards |
|  | Piovesana et al. 2017 | - Digit span backwards |
|  | Van de ven et al. 2017 | - Letter-Number sequencing |
|  | Westerberg et al. 2007 | - Digit span forward and backwards |
| Attention | Cho, Kim & Jung, 2015 | - Visual continous performance test (Total correct) - Visual continous performance test (Reaction time) - Auditory controlled continous performance test (Total correct) - Auditory controlled continous performance test (Reaction time) |
|  | Lin et al. 2014 | - Mental control (WMS-R) - Trail Making Test A - Trail Making Test B |
|  | Piovesana et al. 2017  Piovesana et al. 2017 | - Comprehensive Trail Making Test: Trail 2 - Comprehensive Trail Making Test: Trail 3 - Comprehensive Trail Making Test: Trail 4 - Comprehensive Trail Making Test: Trail 5 - TEA-Ch Sky Search - TEA-Ch Score - TEA-Ch SkySearch DT |
|  | Van de Ven et al. 2017 | - Attention composite (Trail Making Test A, Paced auditory serial addition test and Digit-symbol coding) - D-Kefs TMT (number-letter switching) |
|  | Westerberg et al. 2007 | - Stroop time (sec.) - Stroop raw score - Raven (max 18) - Paced auditory serial addition test - Ruff 2&7 |
|  | Yoo et al. 2015 | - Auditory continuous performance test - Visual continuous performance test - Trail making test composite |
| Processing speed | Lin et al., 2014 | - Trail Making Test A |
|  | Piovesana et al., 2017 | - Symbol Search (WISC-IV) - Coding (WISC-IV) - Colour Naming (D-KEFS: CWI) - Word Reading (D-KEFS: CWI) |
|  | Van de ven et al., 2017 | - Processing speed composite (D-Kefs TMT motor speed condition and Mouse skills task (Neurotask BV)) |
|  | Westerberg et al., 2007 | - Stroop time (sec.) - Paced auditory serial addition test |
|  | Yoo et al., 2015 | - Trail making test (time) |
| General cognition | Åkerlund et al. 2013 | - Barrow Neurological Institute Screen for Higher Cerebral Functions |
|  | Van de Ven et al. 2017 | - Overall cognition composite |
| Visual memory | Lin et al. 2014 | - Visual reproduction (WMS-R) |
|  | Yoo et al. 2015 | - Visual learning test (The Computerized Neuropsychological Test) |
| Verbal memory | Lin et al. 2014 | - Logical memory (WMS-R) - Associated learning (WMS-R) |
|  | Van de ven et al. 2017 | - Rey’s auditory verbal learning test (RAVLT) |
|  | Westerberg et al. 2007 | - Claeson-Dahl test (No. of repetitions) - Claeson-Dahl test (Delayed recall) |
|  | Yoo et al. 2015 | - Verbal learning test (The Computerized Neuropsychological Test) |
| Inhibition | Piovesana et al. 2017 | - D-Kefs (Color-Word Interference) |
|  | Van de ven et al. 2017 | - Stop-signal Task |
|  | Westerberg et al. 2007 | - Stroop time (sec.) - Stroop raw score |
| Flexibility | Lin et al., 2014 | - Trail Making Test B |
|  | Piovesana et al., 2017 | - Comprehensive Trail Making Test: Trail 4 - Comprehensive Trail Making Test: Trail 5 - TEA-Ch SkySearch DT |
|  | Van de ven et al., 2017 | - D-Kefs TMT (number-letter switching) - Flexibility composite (Switch-task, Dual-task, Category fluency switch condition and Trail making test B) |
| Verbal fluency | Van de ven et al., 2017 | - Letter fluency - Category fluency |
| Reasoning | Man et al., 2014 | - Problem solving self-made tests composite (Convergence, Divergence - Comparison, Basic skills and Functional skills) |
|  | Piovesana et al., 2017 | - Tower of London (Total correct) |
|  | Van de ven et al., 2017 | - Tower of London - Reasoning composite (Raven Progressive Matrices and Shipley institute of Living Scale) |
|  | Westerberg et al., 2007 | - Raven Progressive Matrices |
