# Supplementary material for: Computer-based cognitive interventions in acquired brain injury: A systematic review and meta-analysis of randomized controlled trials
Source: PLoS One. 2020 Jul 9;15(7):e0235510. doi: 10.1371/journal.pone.0235510 (PMC7347178; doi:10.1371/journal.pone.0235510)
Supplement: S1 File — (DOCX) [file pone.0235510.s003.docx]

**S1 File. Forest plots and heterogeneity tests of non-significant cognitive domains.**
